# Reversible Control by Vitamin D of Granulocytes and Bacteria in the Lungs of Mice: An Ovalbumin-Induced Model of Allergic Airway Disease

**DOI:** 10.1371/journal.pone.0067823

**Published:** 2013-06-24

**Authors:** Shelley Gorman, Clare E. Weeden, Daryl H. W. Tan, Naomi M. Scott, Julie Hart, Rachel E. Foong, Danny Mok, Nahiid Stephens, Graeme Zosky, Prue H. Hart

**Affiliations:** 1 Telethon Institute for Child Health Research and Centre for Child Health Research, University of Western Australia, Perth, Australia; 2 Department of Microbiology, PathWest Laboratory Medicine WA, Queen Elizabeth II Medical Centre, Nedlands, Western Australia, Australia; 3 School of Veterinary and Biological Sciences, Faculty of Health Sciences, Murdoch University, Perth, Australia; University Hospital Freiburg, Germany

## Abstract

Vitamin D may be essential for restricting the development and severity of allergic diseases and asthma, but a direct causal link between vitamin D deficiency and asthma has yet to be established. We have developed a ‘low dose’ model of allergic airway disease induced by intraperitoneal injection with ovalbumin (1 µg) and aluminium hydroxide (0.2 mg) in which characteristics of atopic asthma are recapitulated, including airway hyperresponsiveness, antigen-specific immunoglobulin type-E and lung inflammation. We assessed the effects of vitamin D deficiency throughout life (from conception until adulthood) on the severity of ovalbumin-induced allergic airway disease in vitamin D-replete and -deficient BALB/c mice using this model. Vitamin D had protective effects such that deficiency significantly enhanced eosinophil and neutrophil numbers in the bronchoalveolar lavage fluid of male but not female mice. Vitamin D also suppressed the proliferation and T helper cell type-2 cytokine-secreting capacity of airway-draining lymph node cells from both male and female mice. Supplementation of initially vitamin D-deficient mice with vitamin D for four weeks returned serum 25-hydroxyvitamin D to levels observed in initially vitamin D-replete mice, and also suppressed eosinophil and neutrophil numbers in the bronchoalveolar lavage fluid of male mice. Using generic 16 S rRNA primers, increased bacterial levels were detected in the lungs of initially vitamin D-deficient male mice, which were also reduced by vitamin D supplementation. These results indicate that vitamin D controls granulocyte levels in the bronchoalveolar lavage fluid in an allergen-sensitive manner, and may contribute towards the severity of asthma in a gender-specific fashion through regulation of respiratory bacteria.

## Introduction

Vitamin D supplementation is currently under evaluation for the treatment of many pathological conditions including allergic and autoimmune diseases characterised by aberrant immunity. For example, there are currently greater than forty clinical trials registered at ClinicalTrials.gov in which the potential for vitamin D supplementation in controlling asthma is being assessed (using vitamin D and asthma as keywords). Skin exposure to the ultraviolet rays of sunlight is the main means by which individuals acquire vitamin D. After exposure to ultraviolet radiation, epidermal 7-dehydrocholesterol undergoes chemical conversion to pre-vitamin D, which is then further isomerised to vitamin D, is bound by the vitamin D-binding protein and relocated to the liver and kidney for sequential hydroxylations to form 25-hydroxyvitamin D (25(OH)D) and 1,25-dihydroxyvitamin D (1,25(OH)_2_D). Immune cells like dendritic cells and macrophages also synthesize 1,25(OH)_2_D through the expression of hydroxylating enzymes like the 1α-hydroxylase (CYP7B1) [1]. Vitamin D is also increasingly acquired through dietary sources particularly in the form of supplements. The best current measure for determining vitamin D sufficiency is 25(OH)D, where deficiency is recognised by many to occur when serum 25(OH)D levels fall below 50 nmol/L [1].

It is currently not clear whether asthmatics have an increased propensity towards vitamin D deficiency than non-asthmatics. Circulating 25(OH)D levels were significantly reduced in children with moderate (mean 25(OH)D = 42 nmol/L) or severe therapy-resistant (mean 25(OH)D = 28 nmol/L) asthma than non-asthmatic children (mean 25(OH)D = 56 nmol/L) [2]. In contrast, other studies have shown that vitamin D deficiency (<50 nmol/L) is more common in non-asthmatics (57%) than asthmatics (48%) [3]. In a case-controlled study of age- and sex-matched adults with and without asthma, there was no significant association between serum 25(OH)D levels and asthma severity [4]. The role of vitamin D in controlling the development of asthma is also controversial. Dietary intake of vitamin D by pregnant mothers has been associated with reduced asthma/wheezing outcomes in their children [5], [6], [7], [8]. In contrast, vitamin D intake in the first few years of life through oral supplementation has been positively associated with asthma in most studies [9], [10], [11]. These observations have led to the somewhat conflicting hypotheses that vitamin D deficiency [12] and oral vitamin D supplementation [13] may both contribute toward the asthma ‘epidemic’. Further recent hypotheses have drawn links between the potential for vitamin D to regulate the gut microbiome and the emergence of asthma and autoimmune disease [14].

There are now a number of studies showing a positive relationship between serum 25(OH)D levels and lung function in both adults [15], [16] and children [2], [17], [18], [19], [20], but again, these relationships are not exhibited by all populations studied [4], [21]. Our own studies have shown that vitamin D deficiency *in utero* affects lung development, where 2 week-old BALB/c mice from vitamin D-deficient mothers had impaired lung volume and function [22]. Current vitamin D status may also moderate asthma, especially in children, although again not all studies are in agreement [18]. Negative correlations of serum 25(OH)D and asthmatic-related parameters like allergen-specific immunoglobulin type-E (IgE), corticosteroid use and airway hyperresponsiveness (AHR) have been observed in pediatric populations from Australia [23], Costa Rica [24], Northern America [3], [17], [20], Qatar [25] and the United Kingdom [2].

While in most studies inverse associations between vitamin D status and asthma-related outcomes have been observed, a direct causal link between vitamin D deficiency and asthma has yet to be established. Treatment with the active vitamin D metabolite, 1,25(OH)_2_D, significantly reduced lung eosinophilia, the severity of the histopathological changes and AHR in mice with allergic airway disease [26], [27], [28], [29]. However, vitamin D deficiency had minimal effects on allergic airway disease induced in C57Bl/6 mice, although these animals became hypocalcemic [30]. In the T helper cell type-2 (Th2) response prone BALB/c mouse, vitamin D deficiency reduced the expression of the vitamin D receptor (VDR) in the lungs of mice with allergic airway disease [31]. Finally, in our own studies, vitamin D deficiency increased the capacity of airway-draining lymph node (ADLN) cells from BALB/c mice with ovalbumin (OVA)-induced allergic airway disease to proliferate and secrete Th2 cytokines in response to *ex vivo* OVA stimulation [32]. However, lung eosinophilia and OVA-specific IgE levels were not modified by vitamin D deficiency.

There is considerable debate about the supra-physiological doses of allergen and adjuvant used in murine models of asthma [33], [34]. We have previously shown that only 1 µg OVA and 0.2 mg of the Th2-skewing adjuvant Aluminium hydroxide (Alum) are necessary to sensitize mice, and with these more realistic and less aggressive levels of sensitization, regulatory effects of genuine immunomodulators were observed [34]. We hypothesised that the dose of allergen used to sensitize mice by previous investigators (including ourselves, [30], [32]) may have masked some of the potential effects of vitamin D deficiency on the severity of allergic airway disease observed in mice. To assess this hypothesis we sensitized mice with 1 µg OVA and 0.2 mg Alum and examined the effects of a ‘lifetime’ of vitamin D deficiency and its reversibility by vitamin D supplementation in adulthood. With the recent hypotheses that vitamin D may modulate microflora to regulate asthma [14], we also examined the effects of vitamin D on commensal bacterial levels in the lungs of mice with allergic airway disease.

## Materials and Methods

### Mice and diet

All experiments were performed according to the ethical guidelines of the National Health and Medical Research Council of Australia and with approval from the Telethon Institute for Child Health Research Animal Ethics Committee. Mice were purchased from the Animal Resources Centre, Western Australia. Female 3 wk-old BALB/c mice were placed on semi-pure diets, which were supplemented with vitamin D_3_ (2280 IU vitamin D_3_/kg, SF05-34, Specialty Feeds, Perth, Western Australia) or were not supplemented vitamin D_3_ (0 IU vitamin D_3_/kg, SF05-033, Specialty Feeds). The non-vitamin D-supplemented diet was also supplemented with 2% calcium (vs 1% for the vitamin D-supplemented diet). At 8 weeks of age, female mice were mated with adult male BALB/c mice maintained on standard mouse chow (Specialty Feeds, containing 2000 IU vitamin D_3_/kg). The dietary doses of vitamin D in the vitamin D-replete diet are similar to those found in standard mouse chow (Specialty Feeds). Offspring born following these matings were maintained on the vitamin D-replete or -deficient diets for the rest of the experiment, except in some experiments where initially vitamin D-deficient mice were switched to the vitamin D-replete diet from 8 weeks of age, or initially vitamin D-replete mice were switched to the vitamin D-deficient diet from 4 weeks of age. Mice were housed under perspex-filtered fluorescent lighting, which emitted no detectable ultraviolet (UV) B radiation as measured using a UV radiometer (UVX Digital Radiometer, Ultraviolet Products Inc., Upland, CA, USA). Mice transgenic for the OVA_323–339_ (ISQAVHAAHAEINEAGR)-specific TCR-αβ (DO11.10) on a BALB/c background were originally purchased from the Jackson Laboratory and bred in-house. DO11.10 mice were used between the ages of 8–12 wks. Expression of OVA_323–339_-specific TCR-αβ on T cells was confirmed as previously described [35].

### Sensitization and challenge of mice with ovalbumin

OVA (Sigma Chemical Company, St Louis, MO, USA) was mixed with an aluminium hydroxide suspension (Alum, Serva, Heidelberg, Germany). This OVA/Alum solution was diluted in 0.9% saline to sensitize and boost mice intraperitoneally (200 µl) with 1 µg OVA and 0.2 mg Alum [34]. Mice were sensitized on day 0 and then boosted again on day 14. On day 21, mice were challenged with a 1% OVA-in-saline (1 mg/ml) aerosol delivered using an ultrasonic nebulizer (UltraNebs, DeVilbiss, Somerset, PA, USA) for 30 min. In some experiments, mice were challenged with OVA on three successive days. In other experiments, mice were sensitized and boosted with 0.02 µg OVA and 4 µg Alum, 1 µg OVA and 0.2 mg Alum, or 5 µg OVA and 1 mg Alum.

### Bronchoalveolar lavage fluid for assessment of inflammatory cells and cytokines

Twenty-four hours after the last aerosol challenge with OVA, BALF was collected as described previously, with a total of 1 ml collected of BALF for each mouse [36]. BALF cells (5×10^5^) were spun onto glass slides using a cytocentrifuge, and differential counts of inflammatory cells performed by staining cells with the DIFF-Quik Stain Set 64851 (Lab Aids, Narrabeen, NSW, Australia) as per the manufacturer's instructions. At least 300 cells were counted for each sample from at least 3 independent fields of view (x100). Levels of IL-4, IL-5, IL-6, IL-10, IL-13, IL-17, IL-33, GM-CSF, IFNγ and TNF in BALF were detected using an enzyme-linked immunosorbant assay (ELISA) as previously described [36], [37] with antibody pairs supplied by BD Biosciences (Franklin Lakes, NJ, USA) or eBiosciences (San Diego, CA, USA).

### Assessing the responsiveness of airway-draining lymph nodes cells

Twenty-four hours after OVA challenge, the posterior mediastinal, tracheobrachial and parathymic lymph nodes (airway-draining lymph nodes, ADLN) were removed, pooled within experimental groups, physically disaggregated and cultured at 10^5^ cells/200 µl/well (12 replicates per treatment) with OVA (10 µg/ml) as described previously [32], [36], [37]. Tritiated-[^3^H]-thymidine incorporation was used as a measure of cellular proliferation when added for the final 24 h of a 72 h culture. Culture supernatants were also obtained at 72 h and tested for the presence of IL-4, IL-5, IL-6, IL-10, IL-13, IL-17, IL-33, GM-CSF, IFNγ and TNF by ELISA as previously described [32], [36], [37].

### Measurement of serum and BALF levels of 25-hydroxyvitamin D (25(OH)D)

Serum and BALF 25(OH)D levels were measured using IDS EIA kits (Immunodiagnostic Systems Ltd, Fountain Hills, AZ) as described by the manufacturer (limit of detection was 5-7 nmol.L^−1^). Serum 25(OH)D levels were measured in female and male breeding animals at 8 weeks of age prior to mating, and also in offspring at 8 weeks of age and 3 days before the respiratory challenge with OVA.

### Measurement of OVA-specific IgE and IgG1 in serum

At day 20 (24 h before the first OVA challenge) of the OVA-induced allergic airway disease model, sera were obtained to test for OVA-specific IgE and IgG1. OVA-specific IgE levels were measured using an *in vivo* passive cutaneous anaphylaxis assay described previously [32], [36]. OVA-specific immunoglobulin type-G1 (IgG1) levels in serum were measured using an ELISA assay as previously described [34]. OVA-specific IgE and IgG1 titres are expressed as log_2_ and log_10_, respectively.

### Histopathological assessment of lung inflammation

Twenty-four h after OVA aerosol challenge, under 10 cm H_2_O pressure, 10% buffered-formalin (in phosphate-buffered saline) was instilled through the trachea into the lungs of euthanased mice for 2 h. Lungs were removed and fixed for a further 4 days with 10%-buffered formalin. Tissue sections were stained according to routine procedures (haematoxylin and eosin (H&E), or Periodic acid Schiff (PAS)). The distribution of peribronchiolar inflammation and perivascular inflammation was assessed by a veterinary histopathologist in a blinded fashion with a score out of four for each assigned, as per the semi-quantitative scoring system in [38], where; 0 = none, 1 = minimal/marginal, 2 = mild, 3 = moderate, 4 = severe inflammation. The peribronchiolar and perivascular scores were added to give a histopathological score for lung inflammation out of eight.

### Measurement of airway hyperresponsiveness

A modified low-frequency forced oscillation technique was used to measure the change in respiratory system input impedance [39]. Briefly, 24 h after the aerosol challenge with OVA or saline only, mice were anaesthetized with xylazine (2 mg/ml; Troy Laboratories, Smithfield, NSW, Australia) and ketamine (40 mg/ml; Troy Laboratories) delivered i.p. at a dose of 0.01 ml/g. Mice were tracheostomized and ventilated (flexiVent, Scireq, Montreal, QC, Canada) at 450 breaths/min with a tidal volume of 8 ml/kg and a positive end expiratory pressure of 2 cm H_2_O. Baseline values were obtained by measuring impedance five times at 1 min intervals. A 90 sec saline aerosol was delivered with an ultrasonic nebulizer (Devilbiss UltraNebs) and impedance was measured five times at 1 min intervals. This was repeated with ½log_10_ incremental doses of methacholine (0.3–100 mg/ml) and the peak response for each parameter was recorded for analysis. The constant-phase model [40] was used to partition impedance into components representing the conducting airways (airway resistance) and the lung parenchyma (tissue damping and tissue elastance).

### Measurement of circulating immune cells and cytokines

At 24 h after OVA aerosol challenge, 250 µl of blood was obtained from mice and immediately added to K_2_EDTA-coated Microtainer tubes (BD, Franklin Lakes, NJ) to prevent clotting. The proportions of circulating eosinophils, neutrophils and basophils within white blood cells was determined using the ADVIA® 120 Haematology System (Siemens Healthcare Diagnostics Inc, Tarrytown, NY. At day 20 (24 h before OVA challenge) of the OVA-induced allergic airway disease model, sera were tested for the presence of GM-CSF by ELISA as previously described [32], [36], [37] using antibody pairs from eBiosciences.

### In vitro antigen-presenting cell assay

Twenty-four h after OVA challenge, ADLN were removed and digested with collagenase type 4 (1 mg/ml, Worthington Biochemical, Lakewood, NJ) and DNAse (0.1 mg/ml, Sigma) for 30 min at 37°C. CD11c microbeads (Miltenyi Biotech) were used with the autoMACS separator (Miltenyi Biotech) to positively select for CD11c+ cells. Flow cytometry was used to determine that the purity of live isolated CD11c+ cells was routinely >90%. The expression of activation markers such as CD86 on the CD11c+ cells was not modified by this positive selection process (data not shown).

CD4+ cells were isolated from peripheral lymph nodes (auricular, axillary, brachial, inguinal, mesenteric and para-aotric) of DO11.10 mice. A CD4+ T cell isolation kit (Miltenyi Biotech, Cologne, Germany) was used to isolate the CD4+ cells (≥ 95% pure, as determined by flow cytometry). Cells were resuspended in RPMI with 10% FCS and 2 µM 2-ME and aliquot into round-bottomed 96-well plates at 10^5^ cells/0.1 ml/well. OVA_323–339_ peptide (Proteomics International, Perth, Australia) was added at a final concentration of 1 µg/ml. CD11c+ cells were added to CD4+ cells from sex-matched mice at varying ratios (from 1∶10 to 1∶1280) in replicates of six. After incubation for 72 h at 37°C in 5% CO_2_, methyl-[^3^H]-thymidine (Amersham Biosciences; 0.25 mCi; 10 µl/well) was added to cultures prior to the harvest of cells at 96 h, with [H^3^]-thymidine incorporation used as the measure of cellular proliferation.

### FACS analysis of immune cells in airway-draining lymph nodes

Twenty-four h after OVA challenge, ADLN were removed from mice, pooled within experimental groups and physically disaggregated. Staining of surface antigens was performed as previously described [35]. An intracellular staining kit (eBiosciences) was used to determine intracellular Foxp3 expression. At least 10,000 cells of interest were collected using the FACS LSRII (BD Biosciences) flow cytometer. Data were analysed using FlowJo software (v9.5.2, TreeStar Inc, Ashland OR, USA).

### Detection of mRNAs in CD11c+ cells from the airway draining lymph nodes

RNA was purified from 10^5^ purified CD11c+ cells, cDNA transcribed and real-time PCR performed as described previously [41], using a Dendritic Cell and Antigen-Presenting Cell RT^2^ profiler PCR array (PAM406Z, 96-wells) to detect gene expression of CCL5, CCL11, CCL19, CCL20, CXCL1 and IL-18RA as described by the manufacturer (Qiagen, Doncaster, VIC, Australia). Fold-change was determined by using the 2^−ΔΔCt^ method specified by the manufacturer by combining results obtained for multiple house-keeping genes.

### Detection of mRNAs in BALF cells

Messenger RNA was extracted from BALF cells (2.5×10^5^), cDNA synthesized and real-time assays performed as previously described using Quantitect Primer Assays (Qiagen) for detection of mRNAs of CYP2R1, CYP27B1, CYP24A1 and the VDR genes with EEF1α used as the house-keeping control [42]. Fold-change was determined by using the 2^−ΔΔCt^ method.

### Determining bacterial loads in lung samples

Twenty-four h after OVA aerosol-challenge the lungs were removed from mice. The right lobe of each lung was minced using a sterile scalpel blade. A 2 mm^3^ sample of the lung was snap-frozen in liquid nitrogen. DNA was extracted from lung samples using the DNeasy Blood and Tissue DNA extraction kit as described by the manufacturer (Qiagen). Universal 16 S rRNA primers (F primer = 5′-TCCTACGGGAGGCAGCAGT-3`; R primer is 5′-GGACTACCAGGGTATCTAATC CTGTT-3` [43]) were used for the detection of bacteria in DNA samples using the Power SYBR Green PCR Master Mix as described by the manufacturer (Applied Biosystems, Calsbad, USA). These primers have been shown to be broadly specific for the detection of bacterial DNA from 34 bacterial species encompassing most bacterial groups [43]. An 18 S rRNA endogenous control including a FAM MGB probe was used as the internal control for this assay using conditions described by the manufacturer (Applied Biosystems). Fold-change was determined by using the 2^−ΔΔCt^ method. BALF samples were also tested for culturable bacteria on routine clinical media, where 10 µl of pooled BALF samples (for each treatment) were plated directly onto 5% horse blood, chocolate and MacConkey agar (all media obtained from PathWest Laboratory Medicine WA Media, Australia) and incubated at 35 °C in enriched CO_2_ (6%). Anaerobic culture was performed on 5% horse blood agar using a jar and AnaeroPack-Anaero® (MGC, Japan) gas pack. Six days of enrichment prior to subculture to horse blood and chocolate agar was also performed using cooked meat broth inoculated with 10 µl of pooled BALF samples. Plates were reviewed at 24, 48 and 120 h incubation for bacteria colonies.

### Statistical analyses

Data were compared using an unpaired two-way student's *t* test using the Prism 5 for Mac OS X statistical analysis program. For analysis of PCR data, fold-change was transformed by log_2_ and then a one-sample *t* test was performed. An up- or down-regulation of a particular gene transcript was considered to be significant with a p-value <0.05.

## Results

### Dietary vitamin D suppresses some aspects of OVA-induced allergic airway disease in male mice, but is limited by allergen dose

**Figure 1:**
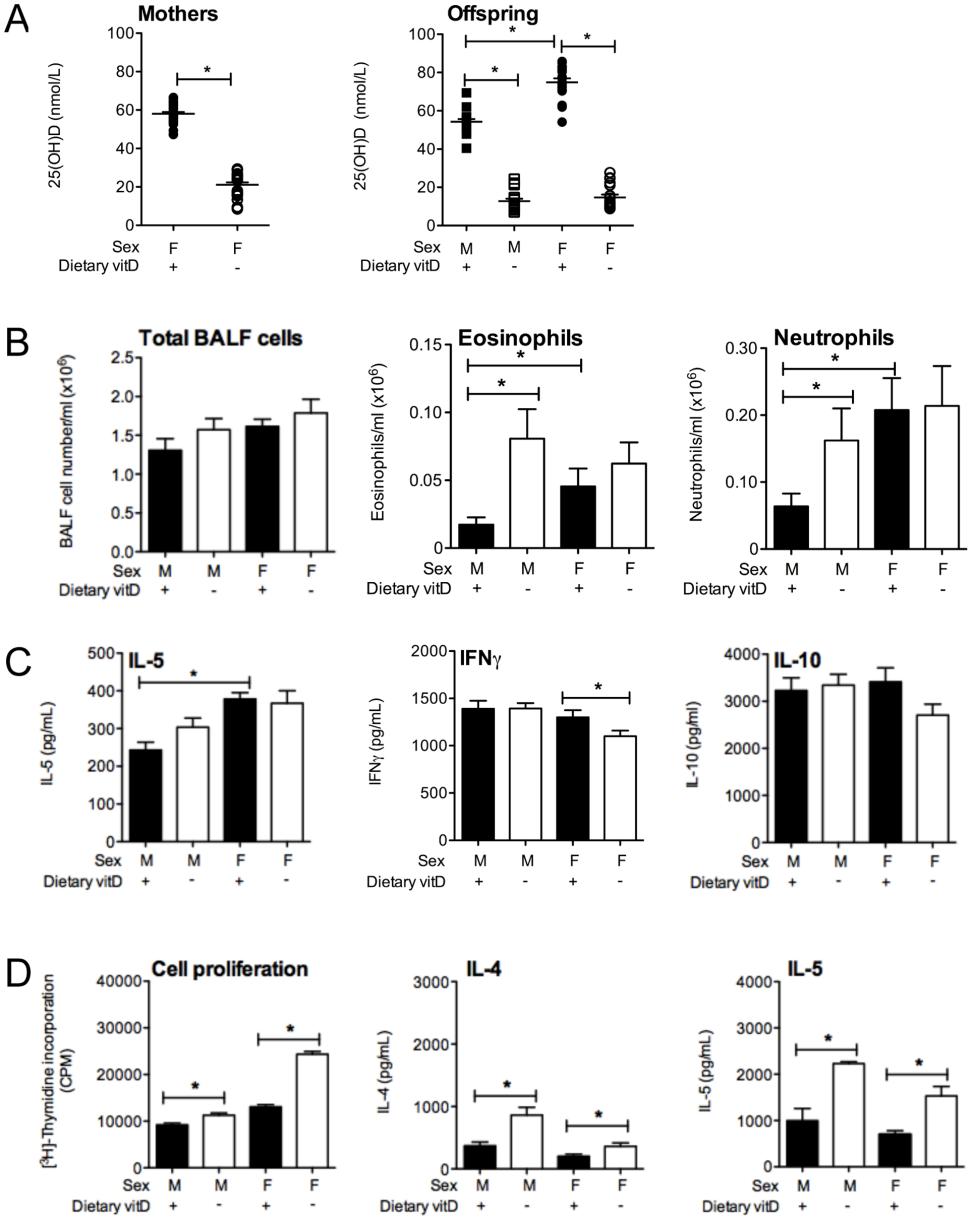
Vitamin D deficiency enhances granulocyte infiltration into the lungs of male but not female mice. In (**A**), serum 25-hydroxyvitamin D (25(OH)D) levels of female BALB/c mice (mothers) fed vitamin D-containing (+) or vitamin D-null (−) diets from 3 weeks of age and were used to produce offspring at 8 weeks of age. Serum 25(OH)D levels for 8 wk-old male and female offspring are also shown. For serum 25(OH)D measurements, data is from ≥17 mice per group. At 8 weeks of age, male and female offspring were sensitized intraperitoneally with 1 µg ovalbumin (OVA) (0.2 mg Aluminium hydroxide (Alum)). Mice were boosted with the same OVA/Alum dose two weeks after the initial sensitization, and one week later their airways challenged with aerosolised OVA (1% in saline), with responses assessed 24 h later. In three repeated experiments (n = 15 mice/treatment); (**B**) the total number of cells as well as the number of eosinophils and neutrophils in bronchoalveolar lavage fluid (BALF) were enumerated; and (**C**) BALF levels of interleukin (IL)-5, interferon (IFN)γ and IL-10 levels were also determined. In (**D**), airway-draining lymph node (ADLN) cells were pooled within treatments (n = 5 mice per treatment) and their proliferation in response to exogenous OVA protein was measured by the addition of tritiated [H^3^]-thymidine for the final 24 h of a 72 h culture. Concentrations of IL-4 and IL-5 were measured in the supernatants (n = 4 wells/treatment) of the cultured ADLN cells. In (**D**), data is shown for a representative experiment of three independent experiments. (mean + SEM, *p<0.05, LOD = limit of detection).

The cumulative effects of a lifetime of vitamin D deficiency were examined, where 8 wk-old offspring born to vitamin D-deficient female mice and maintained on a vitamin D-null diet had mean serum 25(OH)D levels that were <20 nmol/L (n≥17 mice/group, Fig. 1A). Levels of 25(OH)D observed in male mice were significantly less than those observed in the female mice fed the vitamin D-containing diet, as shown previously (n≥17 mice/group, Fig. 1A, [32], [42]). OVA/Alum sensitisation and boost did not modify serum 25(OH)D levels (data not shown). Levels of 25(OH)D observed in male mice fed the standard mouse chow were similar to those reported for male offspring in Fig. 1A. As shown previously [42], male and female mice fed the vitamin D-null diet had reduced circulating 1,25(OH)_2_D levels relative to the mice fed the vitamin D-containing diet, although there was no difference between male and female mice per se [42]. There was no difference in serum calcium levels of mice fed either diet [32], [42].

Vitamin D-replete and -deficient offspring were sensitized and boosted with 1 µg OVA, 0.2 mg Alum. In three experiments (n = 15 mice/treatment), vitamin D deficiency significantly increased the number and percentage of eosinophils and neutrophils in the BALF of male but not female mice (Fig. 1B, data not shown for percentages), 24 h after respiratory challenge with OVA. Indeed, there was no difference in the number and percentage of eosinophils and neutrophils in the BALF of male and female vitamin D-deficient mice. There was no significant increase in BALF cytokine levels with vitamin D deficiency, including IL-4, IL-5, IL-6, IL-10, IL-13, IL-17, TNF and IFNγ (Fig. 1C and data not shown). We are uncertain as to why a significant reduction in IFNγ was observed in the BALF of female vitamin D-deficient mice, relative to their -replete counterparts (Fig. 1C). As observed previously with higher doses of OVA/Alum [32], vitamin D deficiency enhanced the capacity of ADLN cells to proliferate and secrete cytokines like IL-4, IL-5, IL-10 (Fig. 1D, data not shown), but not IL-6, IL-17, TNF or IFNγ (data not shown). As observed previously [44], lung inflammation was more severe in female than male mice, where BALF eosinophil and neutrophil numbers (Fig. 1B) and IL-5 levels (Fig. 1C) were greater in the female relative to the male vitamin D-replete mice.

**Figure 2:**
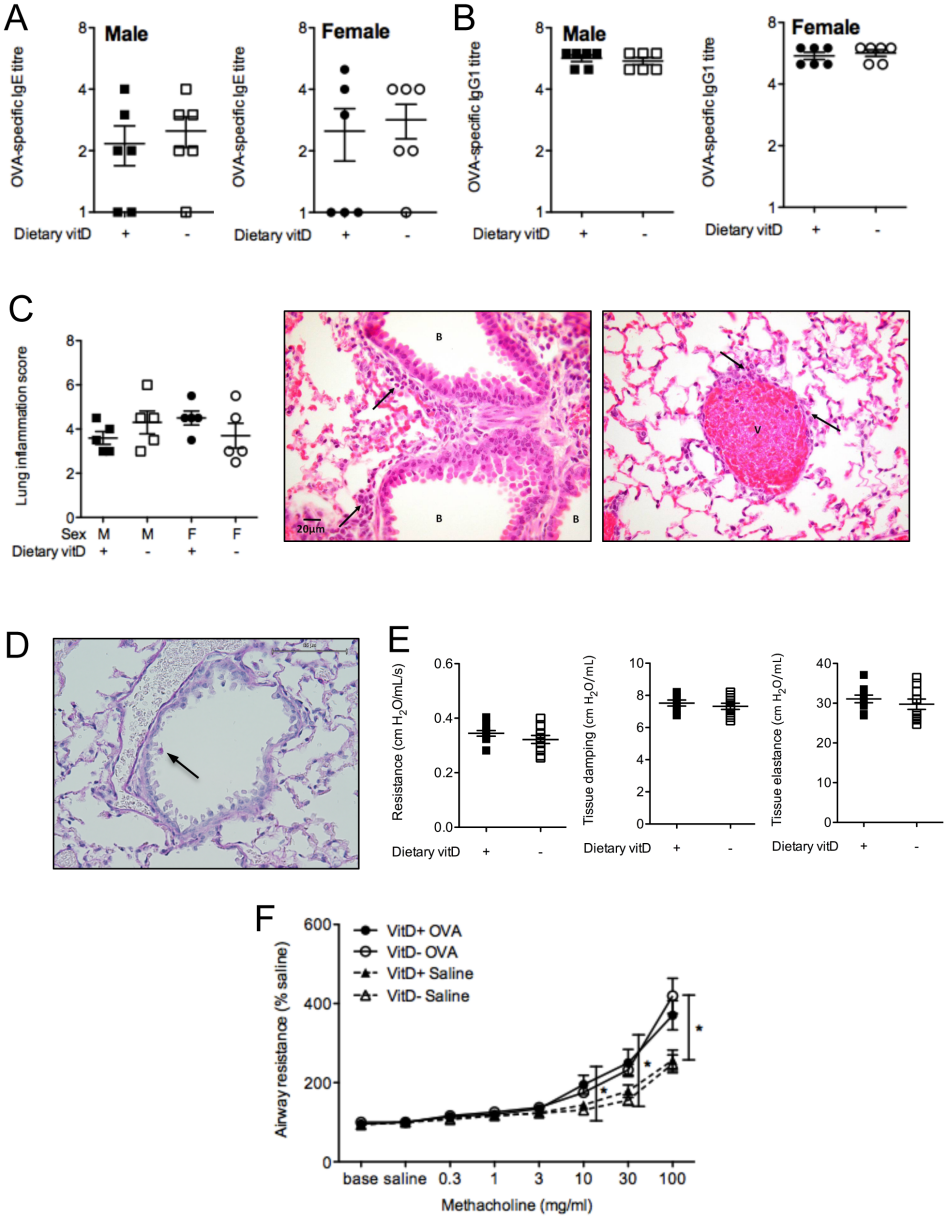
Significant OVA-specific IgE and IgG1, lung inflammation and airway hyperresponsiveness are detected in BALB/c mice. Eight week-old male and female offspring born to vitamin D-replete or -deficient mothers and maintained on the vitamin D-replete or null diets (respectively) were sensitized intraperitoneally with 1 µg ovalbumin (OVA) (0.2 mg Aluminium hydroxide (Alum)). Mice were boosted with the same OVA/Alum dose two weeks after the initial sensitization, and one week later their airways challenged with aerosolised OVA (1% in saline). In (**A**) and (**B**), three days prior to airway challenge, OVA-specific IgE and IgG1 levels, respectively, were measured in the serum of mice (n = 6 per group, with data representative of one experiment of three). In (**C**) the extent of lung inflammation induced 24 h after the airway challenge, is shown as histopathology scores and representative H&E-stained sections exhibiting the average peribronchiolar (score = 1.5; minimal to mild) and perivascular (score = 2; mild) inflammation (indicated by arrows) which mainly consisted of eosinophils (n = 5 per group, 20×magnification; B = bronchioles; V = veins). In (**D**), mucus-secreting (goblet) cells were detected (indicated by arrow and pink staining), with a representative PAS-stained lung section shown. In (**E**), baseline airway resistance and tissue mechanics (tissue elastance and tissue damping) were measured in non-sensitised male offspring mice (n≥7 mice/treatment). In (**F**), airway resistance to increasing concentrations of the bronchoconstrictor methacholine was determined in male offspring mice, 24 h after airway challenge with OVA, or saline only, are shown for OVA-sensitised mice (Baseline = values obtained by measurement of impedance five times at 1 min intervals after stabilization of the mouse on the ventilator; saline = values obtained by measurement of impedance five times at 1 min intervals after a 90 sec saline aerosol;% saline = % increase of saline values for increasing doses of methacholine). Airway resistance is shown as (n≥7 mice/treatment) relative to baseline levels (100%). (mean ± SEM *p<0.05).

**Figure 3:**
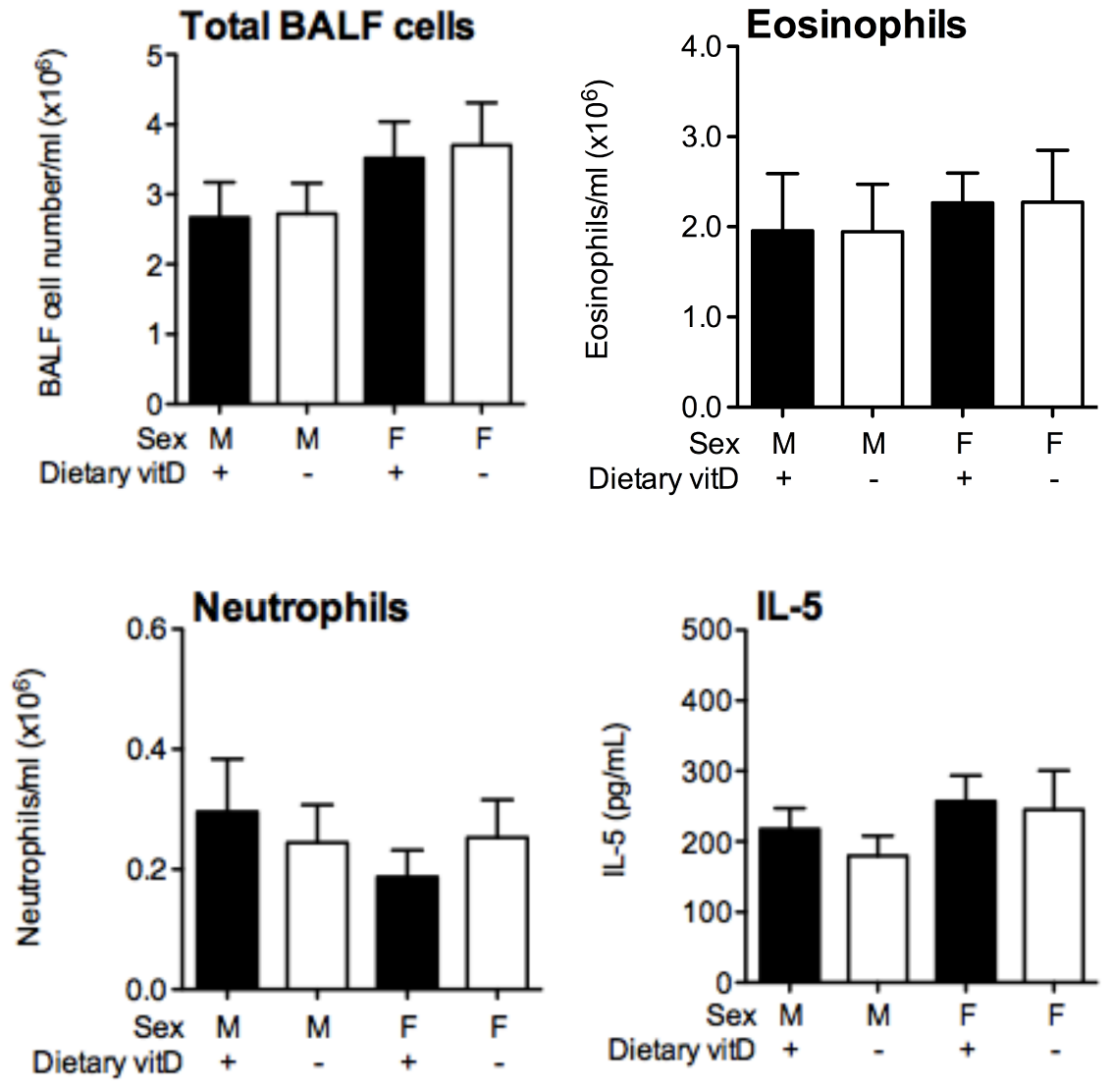
Multiple ovalbumin aerosols overcome the capacity of physiological vitamin D to modulate airway inflammation. Eight week-old male and female offspring born to vitamin D-replete or -deficient mothers and maintained on the vitamin D-containing or null diets (respectively) were sensitized intraperitoneally with 1 µg ovalbumin (OVA) (0.2 mg Aluminium hydroxide (Alum)). Mice were boosted with the same OVA/Alum dose two weeks after the initial sensitization, and one week later their airways challenged with aerosolised OVA (1% in saline) on three consecutive days. The total number of bronchoalveolar lavage fluid (BALF) cells, and the number of eosinophils and neutrophils 24 h after the final OVA challenge are shown along with IL-5 levels. Data is shown as mean+SEM for 10 mice/treatment combined from two independent experiments.

While BALF granulocytes and the responsiveness of ADLN cells were enhanced by vitamin D deficiency (Fig. 1), other important parameters of OVA-induced allergic airway disease were not modified, including serum levels of OVA-specific IgE (Fig. 2A) and IgG1 (Fig. 2B). Histopathological assessment of H&E- and PAS-stained lung sections also revealed no differences between vitamin D-replete and -deficient mice (Fig. 2C). Goblet cell metaplasia was not observed in this model of allergic airway disease (Fig. 2D). Baseline airway responses (airway resistance, tissue elastance or tissue damping) in male mice were not affected by vitamin D deficiency (Fig. 2E). In male mice the airway response (airway resistance) to inhaled methacholine was significantly greater in the OVA-challenge and OVA-sensitized mice than the response observed in saline-challenged but OVA-sensitized mice for the 10–100 mg/ml methacholine challenge doses (Fig. 2F). However, vitamin D deficiency did not significantly modify airway resistance (Fig. 2F), tissue elastance or tissue damping (data not shown) in male mice. In controls from our previous experiments we observed no difference in airway resistance, tissue elastance or tissue damping between (i) naïve mice, (ii) mice sensitised and boosted with OVA/Alum and challenged with saline, and (iii) non-sensitised but OVA-challenged mice [39]. We did not further examine AHR in female mice because we did not initially observe an effect of vitamin D-deficiency on granulocyte numbers in the BALF of female mice. It is clear from the results presented in Fig. 1B and Fig. 2F that the increased BALF granulocyte numbers observed in male vitamin D-deficient mice did not correlate with lung function. In addition, in two experiments (n = 10 mice/treatment) the effect of vitamin D deficiency on BALF granulocyte numbers in male mice was overcome by sequential challenges with OVA (3 daily doses), which enhanced eosinophil numbers in BALF (>100-fold, Fig. 1B; Fig. 3), but had little effect on BALF IL-5 levels (Fig. 1C; Fig. 3). No differences were also observed upon histopathological analysis of H&E-stained lung sections from these mice (data not shown). Multiple OVA challenges also prevented the capacity for dietary vitamin D to suppress the proliferation and Th2-cytokine secreting capacity of ADLN cells *ex vivo* (data not shown). These results indicate that the effects of dietary vitamin D for suppression of OVA-induced allergic airways disease are limited by the dose of allergen used to both sensitize [32] and also challenge mice (Fig. 3).

### Further characterisation of the ‘low dose’ OVA-induced model of allergic airway disease

**Figure 4:**
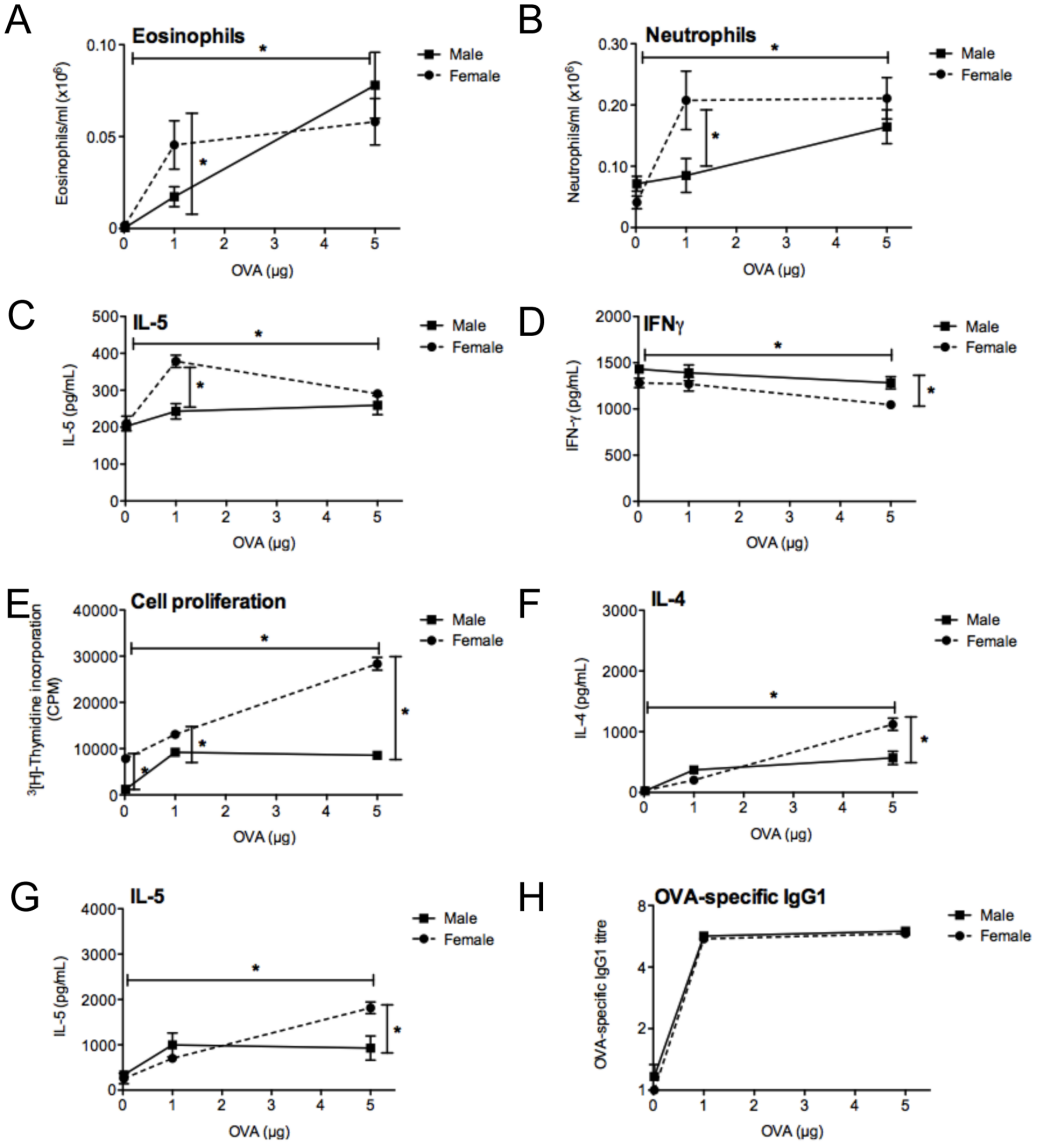
The dose of allergen used to sensitise mice determines the extent of allergic airway disease. At 8 weeks of age, male and female mice were sensitized intraperitoneally with either 0.02 µg ovalbumin (OVA) (4 µg Aluminium hydroxide (Alum)), 1 µg OVA (200 µg Alum), or 5 µg OVA (1 mg Alum). Mice were boosted with the same OVA/Alum dose two weeks after the initial sensitization, and one week later their airways challenged with aerosolised OVA (1% in saline), with responses assessed 24 h later. In three repeated experiments (n = 15 mice/treatment) the number of (**A**) eosinophils and (**B**) neutrophils in bronchoalveolar lavage fluid (BALF) were enumerated and BALF levels of (**C**) interleukin (IL)-5 and (**D**) interferon (IFN)γ were also determined. In (**E**), airway-draining lymph node (ADLN) cells were pooled within treatments (n = 5 mice per treatment; shown are results of a representative experiment) and their proliferation in response to exogenous OVA protein was measured by the addition of tritiated [H^3^]-thymidine for the final 24 h of a 72 h culture. Concentrations of (**F**) IL-4 and (**G**) IL-5 were measured in the supernatants (n = 4 wells/treatment) of the cultured ADLN cells. In (**H**), three days prior to airway challenge, OVA-specific IgG1 levels were measured in the serum of mice. (mean ± SEM, *p<0.05).

To further characterise the ‘low dose’ model of allergic airway disease, we compared responses 24 h after the OVA challenge in mice sensitised and boosted with 0.02 µg OVA and 4 µg Alum, 1 µg OVA and 0.2 mg Alum, or 5 µg OVA and 1 mg Alum. In three experiments (n = 15 mice/treatment), the number of eosinophils (Fig. 4A) and neutrophils (Fig. 4B) was greatest in the BALF of mice sensitised with the highest dose of OVA/Alum (5 µg OVA/1 mg Alum), although neutrophil numbers in the BALF of female mice were comparable at the 1 µg OVA/0.2 mg Alum and 5 µg OVA/1 mg Alum doses (Fig. 4B). The high numbers of neutrophils observed are consistent with our previous studies [32], . IL-5 levels in BALF also increased with increasing dose in the male mice, but were greatest in female mice at the 1 µg OVA/0.2 mg Alum sensitizing dose (Fig. 4C). In contrast, IFNγ levels in the BALF decreased with increasing OVA/Alum dose (Fig. 4D). There was no affect of sensitizing dose on BALF IL-4 or IL-10 levels (data not shown). ADLN cells exhibited an increased capacity to proliferate (Fig. 4E) and produce IL-4 (Fig. 4F) and IL-5 (Fig. 4G) and IL-10 (data not shown) with increasing sensitizing doses of OVA/Alum. For most measures, responses from female mice were greater than that observed in male mice, and this was particularly apparent in the BALF with the 1 µg OVA/0.2 mg Alum dose (Fig. 4A-C). OVA-specific IgG1 levels in serum (measured 3 days before OVA challenge) were maximal with 1 µg OVA/0.2 mg Alum.

### Vitamin D supplementation reverses the effects of a ‘lifetime’ of vitamin D deficiency on BALF granulocyte and ADLN cell responses in male mice

**Figure 5:**
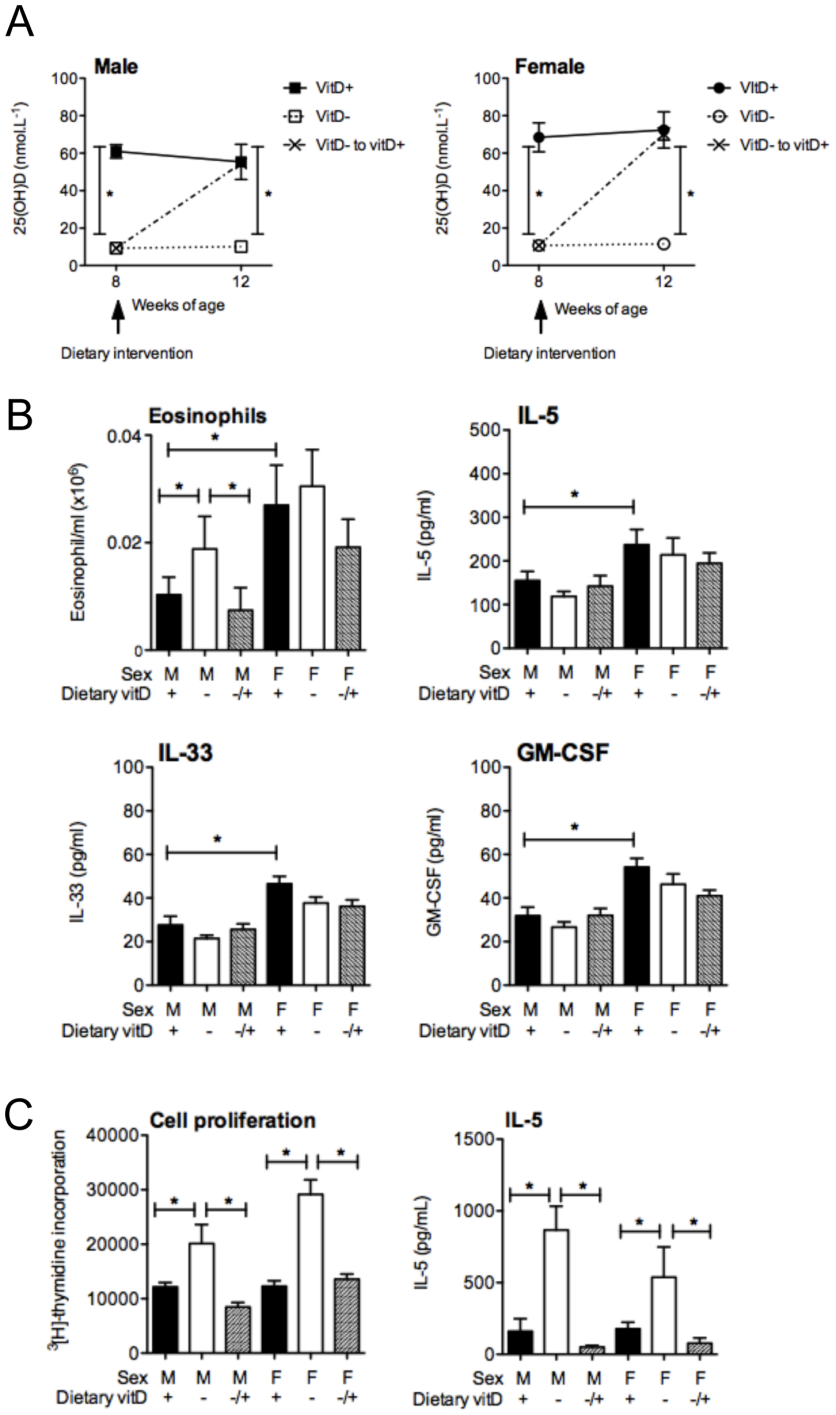
Vitamin D supplementation reverses the effects of deficiency on bronchoalveolar lavage fluid granulocytes and lymph node cell responsiveness. Male and female offspring born to vitamin D-replete or -deficient mothers and maintained on the vitamin D-replete or null diets (respectively), or vitamin D-deficient offspring switched to a vitamin D-replete diet from 8 weeks of age (VitD- to vitD+, −/+), were sensitised at 12 weeks of age intraperitoneally with 1 µg ovalbumin (OVA) (0.2 mg Aluminium hydroxide (Alum)). Mice were boosted with the same OVA/Alum dose two weeks after the initial sensitisation, and one week later their airways challenged with aerosolised OVA (1% in saline). In (A), serum 25-hydroxyvitamin D (25(OH)D) levels prior to and 4 weeks after the dietary intervention (n = 4 mice/treatment, mean ± SEM). In (B), at 24 h after OVA challenge, the number of eosinophils and IL-5, IL-33 and GM-CSF levels in bronchoalveolar lavage fluid (BALF) with data is shown as mean + SEM for ≥15 mice/treatment combined from three independent experiments. In (C), at 24 h after airway challenge with OVA, airway-draining lymph node (ADLN) cells were pooled within treatments (n = 5 mice/treatment) and their proliferation in response to exogenous OVA protein was measured by the addition of tritiated [H^3^]-thymidine for the final 24 h of a 72 h culture. Concentrations of interleukin (IL)-5 were also measured in the supernatants (n = 4 wells per treatment) of the cultured ADLN cells. For (C), data is shown as mean + SEM for a representative experiment of three independent experiments.

In these studies vitamin D deficiency was established in mice from the point of conception. To determine whether the effects of vitamin D deficiency observed in male mice were reversible, 8 week-old vitamin D-deficient mice were fed a vitamin D-containing diet for 4 weeks prior to sensitization. In these mice, serum levels of 25(OH)D increased to that observed in originally vitamin D-replete mice, while mice continued on the vitamin D-null diet exhibited very low levels of serum 25(OH)D (Fig. 5A). In three experiments (n = 15 mice/treatment), increased eosinophil (Fig. 5B) and neutrophil (data not shown) levels in the BALF of originally vitamin D-deficient mice were suppressed by vitamin D supplementation. IL-5 (Fig. 5B) and IL-13 (data not shown) levels in BALF were not altered by vitamin D supplementation. Again, lung inflammation was more severe in female than male mice, where BALF eosinophil numbers and IL-5 (but not IL-13, data not shown) levels were greater in the female relative to the male vitamin D-replete mice (Fig. 5B, also Fig. 1C). In these experiments, BALF levels of IL-33 and GM-CSF were also examined, with no affect of vitamin D, but responses were greater in the female than the male mice (Fig. 5B). The capacity of ADLN cells to proliferate and secrete cytokines (e.g. IL-5, IL-13) was significantly suppressed by vitamin D supplementation in both male and female mice (Fig. 5C, data not shown for IL-13). Supernatant levels of IL-33 were not affected by vitamin D, but were low (<10 mg/ml; level of detection <2 pg/ml). Together, these results indicate that effects of a ‘lifetime’ of vitamin D deficiency, including any epigenetic effects induced *in utero* in male mice are reversible.

### Vitamin D supplementation reduces circulating neutrophil and basophil numbers in male mice with OVA-induced allergic airway disease

**Figure 6:**
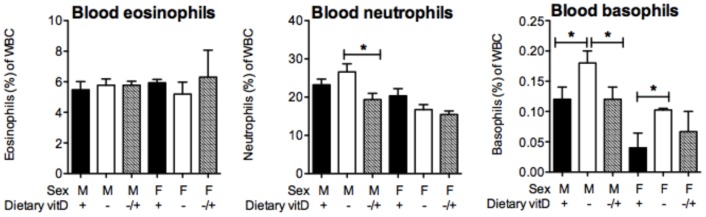
Vitamin D supplementation suppresses circulating neutrophil and basophil levels in male mice. Male and female offspring born to vitamin D-replete or -deficient mothers and maintained on the vitamin D-replete or null diets (respectively), or vitamin D-deficient offspring switched to a vitamin D-replete diet from 8 weeks of age (VitD− to vitD+, −/+), were sensitised at 12 weeks of age intraperitoneally with 1 µg ovalbumin (OVA) (0.2 mg Aluminium hydroxide (Alum)). Mice were boosted with the same OVA/Alum dose two weeks after the initial sensitisation, and one week later their airways challenged with aerosolised OVA (1% in saline). Twenty-four h after the OVA challenge, the proportions of eosinophils, neutrophils and basophils of circulating white blood cells (WBC) is shown as mean + SEM for n = 5 mice/treatment for a representative experiment.

To evaluate whether vitamin D supplementation modulated the migration of granulocytes into the lungs from the blood, circulating levels of eosinophils, neutrophils and basophils were examined 24 h after OVA challenge. While eosinophil numbers were not affected, vitamin D supplementation significantly suppressed the proportions of neutrophils within the white blood cell population in male but not female mice (Fig. 6). In addition, the proportion of circulating basophils was significantly lower in vitamin D-replete mice and was suppressed by vitamin D supplementation of initially vitamin D-deficient male mice (Fig. 6). Circulating levels of GM-CSF were not modulated by vitamin D, when measured 3 days prior to OVA challenge (data not shown), and as reported in Fig. 5B, dietary vitamin D did not affect BALF levels of GM-CSF 24 h post-OVA challenge (data not shown). These results suggest a role for vitamin D in affecting the mobilisation of granulocytes from the bone marrow into the blood with the potential to migrate into inflammatory sites like the lungs of mice with allergic airway disease.

### Vitamin D deficiency induced post-weaning increases BALF granulocyte and ADLN cell responses in male mice

**Figure 7:**
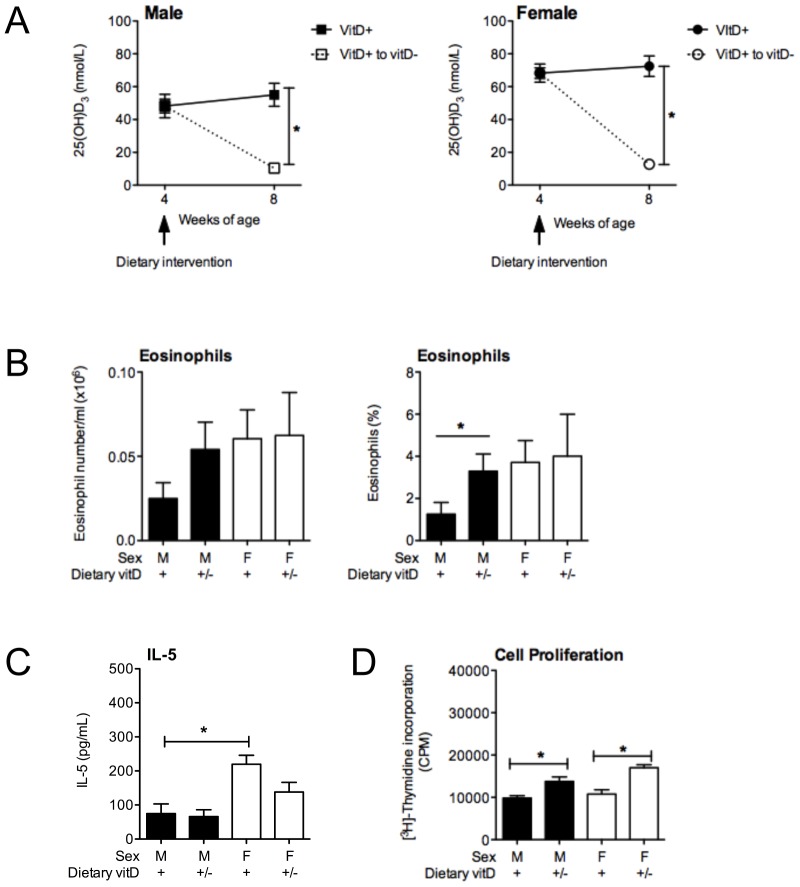
Vitamin D deficiency induced post-weanling enhances granulocyte infiltration into the lungs of male but not female mice. Male and female offspring born to vitamin D-replete mothers were maintained on the vitamin D-replete diets, or switched to a vitamin D-deficient diet from 4 weeks of age (VitD+ to vitD−, +/−). Offspring were sensitised at 8 weeks of age intraperitoneally with 1 µg ovalbumin (OVA) (0.2 mg Aluminium hydroxide (Alum)). Mice were boosted with the same OVA/Alum dose two weeks after the initial sensitisation, and one week later their airways challenged with aerosolised OVA (1% in saline). In (A), serum 25-hydroxyvitamin D (25(OH)D) levels prior to and 4 weeks after the dietary intervention (n = 4 mice/treatment, mean ± SEM). In (B), at 24 h after OVA challenge, the number and percentage of eosinophils and in (C) IL-5 levels in bronchoalveolar lavage fluid (BALF) is shown as mean + SEM for 10 mice/treatment combined from two independent experiments. In (D), at 24 h after airway challenge with OVA, airway-draining lymph node (ADLN) cells were pooled within treatments (n = 5 mice/treatment) and their proliferation in response to exogenous OVA protein was measured by the addition of tritiated [H^3^]-thymidine for the final 24 h of a 72 h culture. For (D), data is shown as mean + SEM for a representative experiment of two independent experiments.

To further confirm that the observed effects of vitamin D were not due to epigenetic effects induced *in utero* or neonatally, vitamin D-replete offspring were fed a vitamin D-deficient diet from 4 weeks of age. Serum 25(OH)D levels were measured four weeks after the dietary intervention, and were significantly reduced to levels (Fig. 7A) previously observed in mice fed the vitamin D-deficient diet throughout life (Fig. 1A, Fig. 5A). Serum 25(OH)D levels remained unchanged for mice fed the vitamin D-replete diet (Fig. 7A). At this time, mice were sensitized and boosted with 1 µg OVA, 0.2 mg Alum. In two experiments (n = 10 mice/treatment), vitamin D deficiency significantly increased the percentage (but not numbers) of eosinophils (Fig. 7B) and neutrophils (data not shown) in the BALF of male but not female mice, 24 h after respiratory challenge with OVA. As before, there was no significant increase in BALF cytokine levels with vitamin D deficiency (e.g. IL-5, Fig. 7C and data not shown for IL-13). Again, vitamin D deficiency enhanced the capacity of ADLN cells to proliferate (Fig. 7D) and secrete cytokines like IL-5 and IL-13 (data not shown). These studies confirm that current (and not past) circulating levels of vitamin D control airway granulocyte and ADLN cell responses in this low-dose model of OVA-induced allergic airway disease.

### Effects of vitamin D deficiency on dendritic cells and regulatory T cells

**Figure 8:**
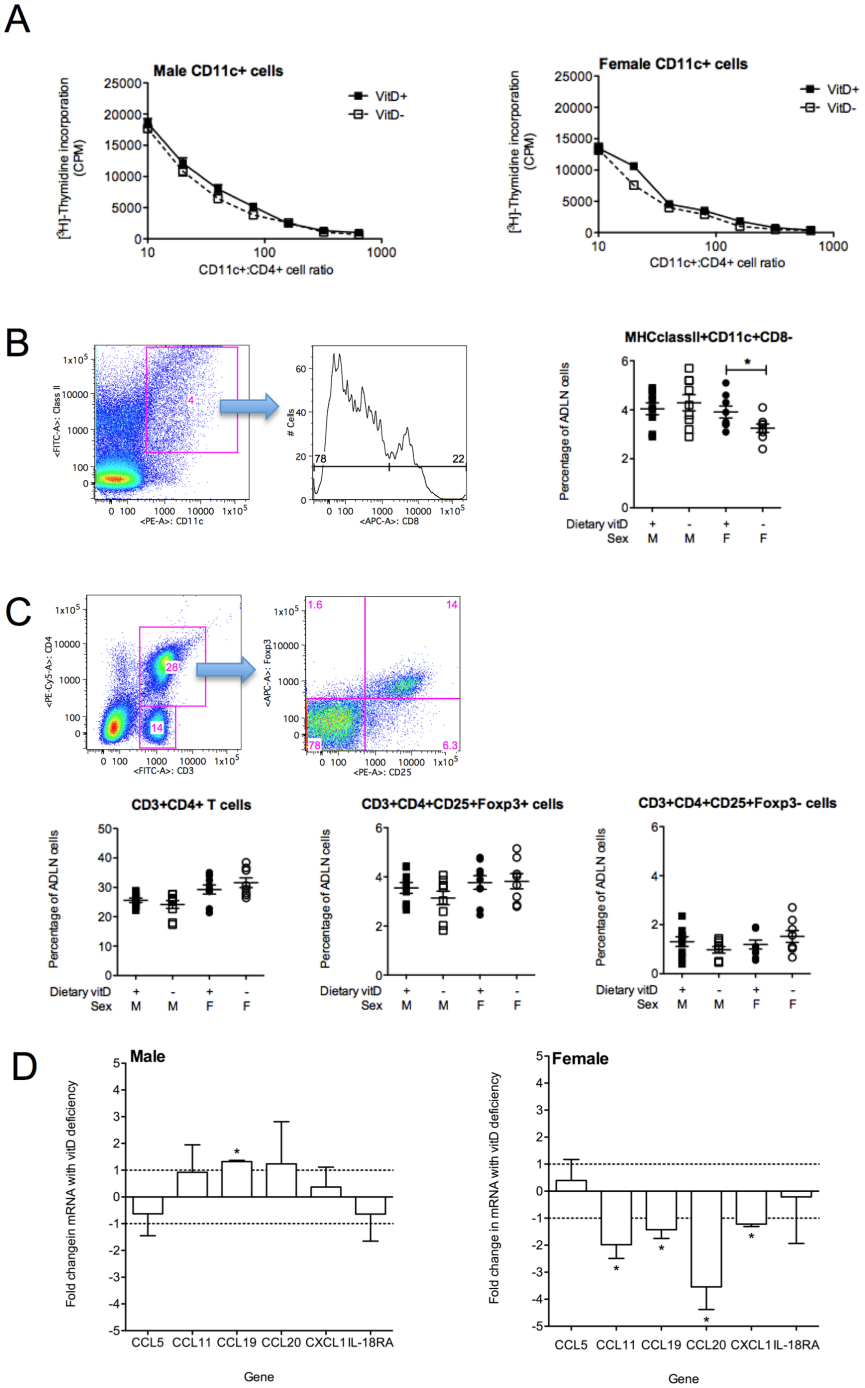
Vitamin D deficiency does not affect the immunogenicity of dendritic cells or the numbers of regulatory T cells in the airway-draining lymph nodes. Eight week-old male and female offspring born to vitamin D-replete or -deficient mothers and maintained on the vitamin D-containing or -null diets (respectively) were sensitized intraperitoneally with 1 µg ovalbumin (OVA) (0.2 mg Aluminium hydroxide (Alum)). Mice were boosted with the same OVA/Alum dose two weeks after the initial sensitization, and one week later their airways challenged with aerosolised OVA (1% in saline). Cells were assessed twenty-four h after OVA challenge. In (A), the capacity of CD11c+ cells isolated from the ADLN to process and present antigen to co-cultured CD4+ T cells from DO11.10 mice was determined by [^3^H]-thymidine incorporation for the final 24 h of 96 h co-culture with 1 µg/ml OVA peptide. Results are shown of a representative of 3 independent experiments (mean±SEM) for CD11c+ cells cultured at various ratios with CD4+ cells. In (B), the FACS gating strategy used to determine the percentage of ClassII+CD11c+CD8− cells in ADLN for 8–9 mice per treatment pooled from 3 independent experiments (mean±SEM). In (C), the FACS gating strategy used to determine the percentage of CD3+CD4+ T cells and CD3+CD4+CD25+Foxp3+ and CD3+CD4+CD25+Foxp3− cells in the ADLN for 8–9 mice per treatment pooled from 3 independent experiments (mean±SEM). In (D), the expression of CCL5, CCL11, CCL19, CCL20, CXCL1 and IL-18RA mRNAs in CD11c+ cells isolated from pooled ADLN of ten mice per treatment with results shown from 3 independent experiments (mean+SEM). (*p<0.05).

Vitamin D induces tolerance through effects on dendritic cells (DCs) and regulatory T cells [1], [45]. In addition to the effects of vitamin D deficiency on BALF granulocytes in male mice, we also observed significantly (p<0.05) increased numbers of ADLN cells in male (vitD+ 7.3±0.6×10^6^; vitD− 1.0±0.1×10^7^ cells/mouse, mean±SEM) but not female (vitD+ 8.0±1.7×10^6^; vitD− 9.3±1.8×10^6^ cells/mouse, mean±SEM) vitamin D-deficient mice, 24 h post-OVA challenge (n = 8–9 mice/treatment pooled from 3 independent experiments). Vitamin D deficiency did not affect immunogenicity of DC from the ADLN, where CD11c+ cells purified from the ADLN of vitamin D-deficient or -replete mice had equivalent capacity to induce the proliferation of CD4+ cells from OVA-TCR transgenic mice (DO11.10) (Fig. 8A). Reduced numbers of MHC classII+ CD11c+CD8− DCs were observed in the ADLN of female but not male vitamin D-deficient mice (Fig. 8B). Migratory DCs express low levels of CD8 [46], and thus it is possible that vitamin D deficiency compromises DC migration in female mice. In further analysis of cell populations from the ADLNs, we did not observe any differences in the number of regulatory T cell (CD4+CD25+Foxp3+) or effector T cell (CD4+CD25+Foxp3−) populations in vitamin D-replete or -deficient mice (Fig. 8C).

DC production of chemokines may affect the migration of granulocytes into the respiratory tract [47]. We hypothesised that chemokine production by airway-associated DCs may have affected the migration of granulocytes during vitamin D deficiency in male, but not female mice. We assessed the expression of mRNAs of a number of chemokines in purified CD11c+ cells, including CCL5 (Rantes), CCL11 (Eotaxin), CCL19 (MIP-3β), CXCL1 (KC) and the receptor for IL-8 (IL-8RA). Increased expression of CCL19 mRNA was observed in the CD11c+ cells from male vitamin D-deficient mice, when compared to their vitamin D-replete counterparts, while vitamin D deficiency down-regulated the expression of mRNAs of CCL11, CCL19, CCL20 and CXCL1 in CD11c+ cells from female mice (Fig. 8D). Thus vitamin D deficiency differentially affects the expression of CCL19 mRNA by ADLN DC in male and female mice.

### Vitamin D deficiency enhances the expression of mRNAs of vitamin D-related molecules by BALF cells

**Figure 9:**
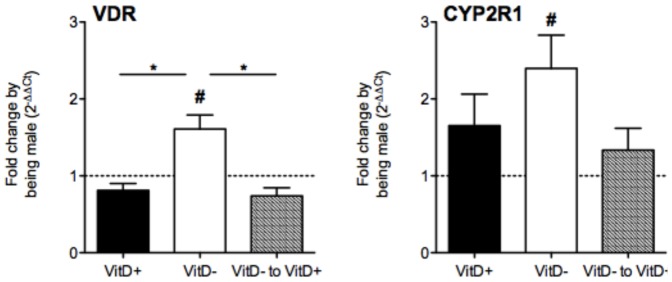
Vitamin D deficiency increases the expression mRNAs of genes encoding the vitamin D receptor and 25-hydroxylase enzyme in bronchoalveolar lavage cells of male mice. Male and female offspring born to vitamin D-replete or -deficient mothers and maintained on the vitamin D-replete or null diets (respectively), or vitamin D-deficient offspring switched to a vitamin D-replete diet from 8 weeks of age (VitD− to VitD+), were sensitised at 12 weeks of age intraperitoneally with 1 µg ovalbumin (OVA) (0.2 mg Aluminium hydroxide (Alum)). Mice were boosted with the same OVA/Alum dose two weeks after the initial sensitisation, and one week later their airways challenged with aerosolised OVA (1% in saline). Shown are relative levels of mRNA of the vitamin D receptor and 25-hydroxylase (CYP2R1) genes induced by being male in bronchoalveolar lavage (BALF) cells (n≥7 mice per group with data as shown as mean + SEM, *p<0.05 between groups, #p<0.05 relative to levels in female mice).

The expression of mRNAs encoding the vitamin D receptor (VDR), and enzymes controlling the production of 25(OH)D (CYP2R1) and 1,25(OH)_2_D (CYP27B1) and the breakdown of 1,25(OH) _2_D (CYP24A1) were examined in BALF cells from vitamin D-replete, -deficient and -deficient mice supplemented with vitamin D, 24 h after respiratory challenge with OVA. CYP27B1 and CYP24A1 gene expression was not detected in BALF cells, indicating that at this time, these cells are not producing detectable quantities of the enzymes that make and break down 1,25(OH)_2_D. In contrast, VDR (mean C_T_-values = 28.9) and CYP2R1 (mean C_T_-values = 33.0) mRNA levels were significantly increased in BALF cells from vitamin D-deficient male mice (relative to female mice), and this increase was reversed by vitamin D supplementation (Fig. 9).

### Vitamin D deficiency enhances the burden of bacteria in the lungs of male mice with OVA-induced allergic airway disease

**Figure 10:**
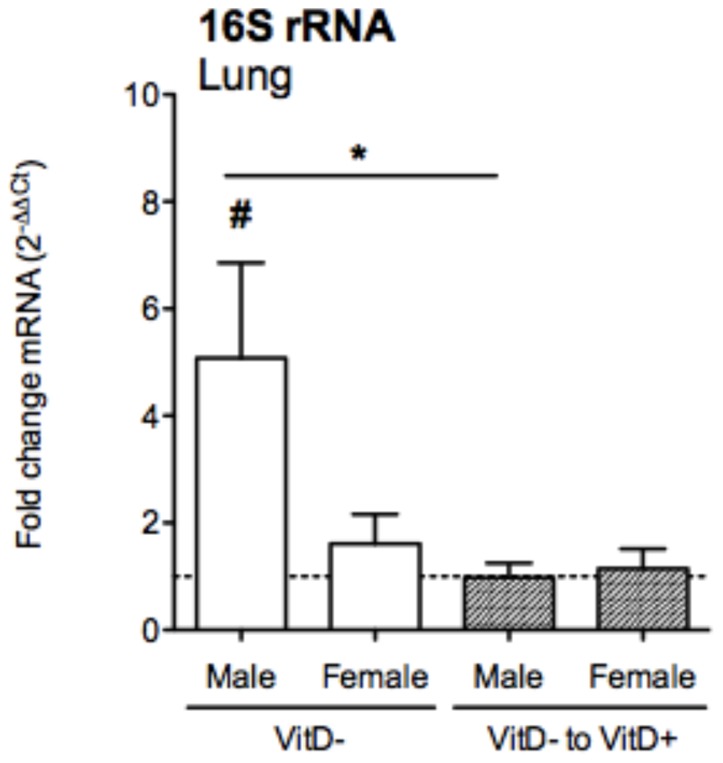
Vitamin D deficiency increases bacterial load in the lungs of male mice with allergic airway disease. Male and female offspring born to vitamin D-replete or -deficient mothers and maintained on the vitamin D-replete or null diets (respectively), or vitamin D-deficient offspring switched to a vitamin D-replete diet from 8 weeks of age (VitD- to VitD+), were sensitised at 12 weeks of age intraperitoneally with 1 µg ovalbumin (OVA) (0.2 mg Aluminium hydroxide (Alum)). Mice were boosted with the same OVA/Alum dose two weeks after the initial sensitisation, and one week later their airways challenged with aerosolised OVA (1% in saline). Shown are bacterial DNA levels induced relative to levels in vitamin D-replete, sex-matched mice as measured in the lungs of mice using a PCR with redundant primers for detection of bacterial 16 S rRNA gene 24 h after the OVA aerosol (mean + SEM for ≥12 mice/treatment from three independent experiments, *p<0.05 between groups, #p<0.05 relative to levels in vitamin D-replete mice).

Plausible links between vitamin D, commensal bacteria and asthma have already been postulated [14]. We hypothesised that dietary vitamin D may co-regulate both commensal bacterial levels and inflammation in the lungs of mice with allergic airway disease. Using universal 16 S rRNA primers to measure commensal bacterial levels, a significantly increased bacterial burden was detected in the lungs (mean 16 S rRNA C_T_-values = 29.9) of vitamin D-deficient male mice (Fig. 10). This increased burden was significantly reduced by vitamin D supplementation to levels observed in vitamin D-replete mice. Vitamin D deficiency and subsequent supplementation did not modify bacterial loads in the lungs of female mice (Fig. 10). Pooled BALF samples (within treatments) were inoculated onto 5% horse blood, chocolate and MacConkey agar for growth in enriched CO_2_ and also anaerobically on blood agar to test for the presence of known (or other) bacterial respiratory pathogens in mice, including *Pseudomonas aeruginosa, Pasteurella pneumotropica*, *Corynebacterium kutscheri*, *Staphylococcus aureus, S. epidermidis, Streptococcus* spp., *Klebsiella* spp. and *Proteus mirabilis* [48]. However, no bacteria were cultured from these samples, even with anaerobic incubation and 6 days enrichment in cooked meat broth prior to subculture to horse blood and chocolate agar. These results suggest that it is the non-culturable microbiome that is altered by vitamin D and point to an important role for commensal bacteria in determining the intensity of lung inflammation in male vitamin D-deficient mice.

## Discussion

Here we provide new evidence for a direct causal link between vitamin D deficiency and asthma. We used a ‘low dose’ model of allergen sensitization to determine whether vitamin D deficiency altered the severity of allergic disease in BALB/c mice. We showed that deficiency in circulating vitamin D levels increased granulocyte numbers and commensal bacterial levels in the lungs of male mice with OVA-induced allergic airway disease. Furthermore, the capacity for ADLN cells to proliferate and secrete Th2 cytokines was exacerbated by vitamin D deficiency in both male and female mice. These effects were reversed by vitamin D supplementation, or induced by post-weaning vitamin D deficiency, suggesting that recent supplementation and ‘current’ vitamin D status are more important for their regulation than long-lived epigenetic modifications established by *in utero* vitamin D deficiency. These results highlight the potential contribution that therapies involving vitamin D supplementation may have for the control of the lung inflammation that occurs in asthmatics, where vitamin D may elicit its protective effects through regulation of the respiratory microbiome.

The lung contains quantities of bacterial communities comparable to the upper respiratory tract, where colonization by certain bacterial species can promote lung inflammation [49]. For example, specific bacterial species (eg. *Chlamydia pneumoniae*) have been linked with asthma exacerbations (reviewed in [49]) and the lungs of asthmatic individuals are more likely to colonised by proteobacteria [50]. From the results of our studies, we propose that in male mice circulating levels of 25(OH)D may control bacterial levels and thus the infiltration of granulocytes into the lungs. The mechanism for this is unclear but may involve sex differences in the regulation of antimicrobial peptides and antibacterial defence systems. A link between sex, antimicrobial peptides and asthma has already been established where single nucleotide polymorphisms in the ß-defensin-1 gene were associated with the diagnosis of asthma in female but not male subjects [51]. In addition, serum 25(OH)D levels in children of the West Australian Pregnancy Cohort (Raine Study) at both 6- (n = 989) and 14-years (n = 1380) of age, were negatively associated with concurrent allergic phenotypes, whereupon sex stratification revealed that this association was restricted mainly to boys [23]. Our analysis of OVA-induced allergic airway disease in vitamin D-deficient mice also reflects a similar male bias. In other studies, rhinovirus infection, now known to be strongly associated with asthma exacerbations [49], occurred more frequently in male than female infants hospitalized for respiratory infections [52]. Furthermore, increased oral corticosteroid use was observed in vitamin D-deficient boys, relative to vitamin D-replete boys of the Childhood Asthma Management Program [53]. Finally, male mice are more susceptible to respiratory infection with some (but not all) bacteria, including *Mycobacterium* species (reviewed in [54]). In the current studies, vitamin D deficiency did not result in enhanced detectable colonisation by ‘culturable’ and known murine respiratory pathogenic bacteria such as *Mycobacterium* species or *Pasteurella pneumotropica* in the lungs of male mice. In future studies, we will examine how vitamin D deficiency differentially affects the non-culturable bacterial microflora of the lungs of male and female mice through deep-sequencing methods such as 454 pyro-sequencing.

There is already substantial evidence linking the protective effects of antimicrobials for dampening the severity of allergic airways disease in mice. An example is cathelin-related antimicrobial peptide (CRAMP), the mouse homologue of human cathelicidin. Cells that express cathelicidins include neutrophils, macrophages, mast cells and epithelial cells (reviewed in [55], [56]). The expression of CRAMP is induced in the lungs during infection with Gram-negative bacteria [57], which rapidly disseminate in knockout mice that do not express CRAMP [58]. Neutrophil influx is delayed early during respiratory infection with Gram-negative bacteria [58]. Somewhat paradoxically, inhaled glucocorticoids, often a very effective treatment of asthma, decreased the expression of CRAMP, increasing the number of internalized *P. aeruginosa* and lung histopathology in infected mice with OVA-induced allergic airway disease [59]. In mice with OVA-induced allergic airway disease, CRAMP protein is not detectable by western blotting of cells in BALF but is detected with further infection with *P. aeruginosa* [57].

Alternatively, the inability of vitamin D to modulate granulocytes and bacteria in the lungs of the female mice may have been due to hormonally-regulated control mechanisms. In particular, there are known interactions between estrogen and vitamin D. A functional synergy has been proposed between 1,25(OH)_2_D and 17-β-estradiol, mediated through estrogen receptor-α, to affect expression of the VDR, vitamin D-inactivating enzyme (CYP24A1) and vitamin D-binding protein [60], [61]. T cells from female subjects with multiple sclerosis have reduced expression of CYP24A1 transcripts [61]. In addition, CD4+ T cells and macrophages from female subjects had an increased ability to take-up the vitamin D-binding protein [61]. Active vitamin D (1,25(OH)_2_D) more potently induces CD4+CD25+Foxp3+ (regulatory) T cells from PBMCs of female than male subjects with multiple sclerosis [61]. In mice with autoimmune encephalomyelitis, the expression of the VDR in the spinal cords was potentiated by estrogen treatment [60]. The cumulative effects of estrogen on vitamin D metabolism and immune cell function highlights how females may be more resilient to the effects of vitamin D deficiency than males.

Along with increased granulocytes in the BALF of vitamin D-deficient male mice, we also observed significant increases in the expression of mRNAs of the VDR and CYP2R1 genes in BALF cells (levels of CYP27B1 and CYP24A1 mRNAs were undetectable). These results may reflect changes in the populations of BALF cells by in-coming cells (eg. granulocytes), which may have enhanced expression of the VDR and CYP2R1 mRNAs relative to other cells. Alternatively, increased expression of the VDR and CYP2R1 may result from a homeostatic response of BALF cells to counter the effects of vitamin D deficiency. We also have previously reported that the reduced 25(OH)D levels in male mice may be due to increased turnover of 25(OH)D as male mice have increased renal expression of mRNAs of the enzyme that makes 1,25(OH)_2_D (CYP27B1) [42]. Our current study adds to the accumulating evidence [42], [60], [61] that vitamin D metabolism and effects of vitamin D on immunity and susceptibility for infection, differs in a sex-dependent fashion.

Viral infection is now commonly thought to induce asthma attacks (reviewed in Brar et al 2012). Vitamin D may reduce the rates of asthma exacerbation by enhancing innate immune mechanisms like antimicrobial peptide production to promote better control of viral infection in asthmatics (reviewed in [1]). Visits to hospital emergency departments and/or hospitalization rates (measures of asthma exacerbation) are associated with reduced circulating levels of serum 25(OH)D in asthmatic children [2], [17], [24], [62], [63]. In addition, first trimester maternal [64] and cord blood [65] levels of 25(OH)D were associated with a reduced risk of respiratory infection in infants at three or twelve months of age, respectively. Recent clinical trials show that this increased risk may be ablated by supplementation with vitamin D. In a randomized trial, when school children were supplemented with 1200 IU/day vitamin D, both the incidence of influenza A infections and the number of asthma attacks in children previously diagnosed with asthma were significantly reduced [66]. These studies indicate that vitamin D supplementation may be a promising strategy for the prevention of asthma exacerbations potentially by reducing the risk for respiratory infection. In future studies, it will be interesting to determine whether increased lung bacterial loads in male vitamin D-deficient mice predispose them towards more severe asthma exacerbations upon viral challenge.

The increased migration of granulocytes into the airways of vitamin D-deficient male mice may have been mediated through enhanced expression of chemokines like CCL19 (MIP-3β) by airway-associated DCs. CCL19 acts through CCR7 [67], which is expressed by a subset of neutrophils [68], eosinophils [69] and basophils [70]. The increased eosinophils and neutrophils observed in the BALF of vitamin D-deficient male mice were also accompanied by enhanced circulating levels of basophils, which have very recently been linked with commensal flora levels in mice [71]. It should be noted that the ADVIA 120 hematology counter may underestimate the number of basophils in specimens and does not perform as well as other methods like flow cytometry for determining circulating basophil numbers [72]. However, serum 25(OH)D levels have previously been negatively associated basophils [23] and other circulating granulocytes including eosinophils [23], [24] and neutrophils [23]. Increased circulating basophils occurred in mice treated orally with antibiotics, which also exhibited exaggerated basophil-mediated Th2 responses and allergic inflammation [71]. In our studies, circulating basophil numbers also correlated with capacity of ADLN cells to proliferate and produce IL-5 in both male and female mice with allergic airway disease. In other studies, a lack of an observed effect of serum 25(OH)D observed upon sputum granulocyte numbers may been masked by anti-inflammatory therapies used to treat children with moderate or severe, therapy-resistant asthma [2].

The observed, potentially beneficial effects of dietary vitamin D on BALF granulocyte numbers in male mice were overcome by increasing the burden of allergen used to sensitize mice [32] or the cumulative quantity used to challenge mice, indicating that excessive allergen exposure may overpower any advantage obtained from having sufficient vitamin D. Because of the these observations, we suggest that the suppressive effects of vitamin D on granulocyte numbers in the BALF of male mice (challenged once with OVA) were unlikely to be due to the introduction of LPS through the aerosol challenge of mice with OVA. We also examined the effects of vitamin D deficiency upon mice intranasally sensitized with 10 daily doses of 25 µg house dust mite (*Dermatophagoides pteronyssinus*) extract (Greer Laboratories). Vitamin D deficiency did not exacerbate BALF granulocyte numbers in either male or female house dust mite-sensitized mice, when cells were examined 24 h after the final intranasal treatment (data not shown). We hypothesize, that like the ‘high dose’ sensitization models for OVA-induced allergic airway disease (eg. 20 µg OVA, 4 mg Alum, [32]), the quantities of house dust mite extract used to sensitize mice need to be further optimised before any regulatory effect of dietary vitamin D can be observed. We also suggest that utilising ‘low dose’ allergen sensitization may be more appropriate to model responses in humans and have previously used a similar ‘low dose’ model to show that ultraviolet radiation can modulate OVA-induced allergic airway disease in pre-sensitized mice [34].

In conclusion, our findings reiterate the importance of maintaining sufficient vitamin D levels for better lung health, and also highlight why it is essential to examine potentially differing pathological responses in both males and females.
